# Drug development targeting degeneration of the basal forebrain cholinergic system: its time has come

**DOI:** 10.1186/s13024-023-00663-y

**Published:** 2023-10-04

**Authors:** John J. Alam, Ralph A. Nixon

**Affiliations:** 1 CervoMed Inc., 20 Park Plaza, Suite 424, Boston, MA 02116 USA; 2 Center for Dementia Research, Nathan S. Kline Institute for Psychiatric Research, Orangeburg, NY USA; 3 Departments of Cell Biology and Psychiatry, NYU Langone Medical Center, NYU Neuroscience Institute, New York, NY USA

**Keywords:** Cholinergic degeneration, Basal forebrain, Rab5, Neflamapimod, p38α, Alzheimer’s disease, Dementia with Lewy bodies

Recent advances in understanding the pathogenic mechanisms underlying basal forebrain cholinergic (BFC) neuronal degeneration and MRI-based studies provide insight on the contribution of such degeneration at various stages of Alzheimer’s disease (AD) and related dementias and have renewed interest in disease-modifying approaches to treat BFC degeneration. Herein we comment on two recent related publications [1, 2], by our respective academic and industrial teams, describing a major translational step forward towards therapy to treat BFC degeneration in the basal forebrain that we believe argues that the time has come for the field to include novel therapeutics development in this arena as a major focus as we look beyond amyloid beta.

The pharmacological approach described in the two publications was based on a hypothesis that treating cholinergic degeneration would be achieved with an oral drug called neflamapimod that inhibits p38α kinase, an enzyme known to regulate activity of a central player in the pathogenic process underlying BFC degeneration, the endosome-associated protein Rab5 (Fig. 1). The first part of the primary publication [1] describes a vehicle-controlled study in Ts[Rb(12.17^16^)]2Cje (Ts2) mice, a model of Down Syndrome (DS) and early-onset AD (i.e., with adult-onset early endosomal pathology and cholinergic degeneration in the basal forebrain [3]). In these mice, 4 weeks treatment with neflamapimod reduced abnormally elevated Rab5 activity in the brain, reversed both the Rab5-dependent endosomal pathology and the cholinergic degenerative process in the basal forebrain, and corrected behaviors associated with the cholinergic system. The second part of the same publication describes a hypothesis-generating clinical trial in patients with dementia with Lewy bodies (DLB), where BFC degeneration is prominent. A total of 91 participants, all receiving background acetylcholinesterase inhibitor (AChEI) therapy, were randomized 1:1 between neflamapimod 40mg or matching placebo capsules (twice-daily if weight < 80kg or three-times-a-day, TID, if ≥ 80kg). A dose-dependent response was seen in the study, with the lower daily dose showing minimal to no clinical activity while at the 40mg TID dose level there were significant improvements relative to placebo in the primary outcome measure, a cognitive-test battery evaluating attention and executive function, in the Clinical Dementia Rating Sum-of-Boxes (CDR-SB; measuring dementia severity), and on the Timed Up and Go (TUG; measuring functional mobility). Importantly, in the discussion, based on the scientific literature, the authors connected the outcome measures that responded to neflamapimod treatment to the BFC system.

**Fig. 1:**
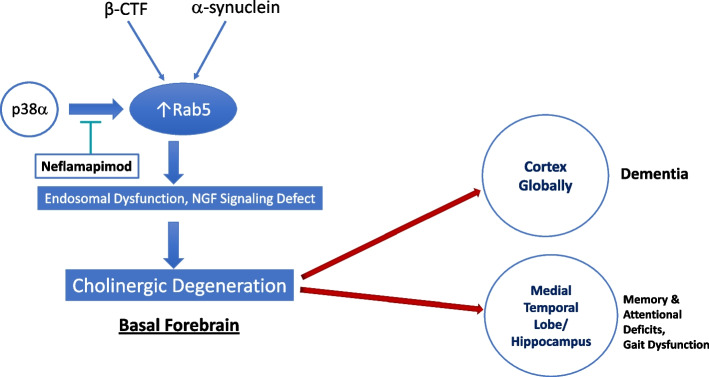
β-CTF and/or α -synuclein induce Rab5-positive (early) endosomal swelling and dysfunction, which leads to a defect in endosome-mediated nerve growth factor (NGF) signaling. The resulting loss of trophic support provided by NGF leads to degeneration of cholinergic neurons in the basal forebrain, that downstream leads to dysfunction and eventual neurodegeneration in the cortex. Neflamapimod through inhibiting the kinase activity of p38α (an activator of Rab5) reduces Rab5 activity and reverses the endosomal pathology and cholinergic neuronal loss in the basal forebrain

In the second publication [2], the same clinical data were stratified by the presence or absence of AD co-pathology, resulting in an analysis that strengthens the conclusions regarding the clinical effect and defines the magnitude of the effect specific to the cholinergic system. The motivation for this analysis was the recognition, based on recent literature, that clinical symptoms in DLB patients without AD co-pathology are due to BFC degeneration while patients with AD co-pathology have extensive cortical atrophy, particularly in the medial temporal lobe, contributing to their symptoms and potentially limiting their response to cholinergic-directed therapies. Accordingly, after the clinical study was completed (i.e., post-hoc), pre-treatment levels of plasma ptau181, a biomarker for the presence of AD co-pathology, were measured in the study participants. In the 16-week treatment period, the 54% of participants with normal baseline plasma ptau181 showed significant improvement with neflamapimod 40mg TID over placebo, with Cohen’s *d* effect size at ≥ 0.7 (*i.e.,* moderate-to-large treatment effects), in tests of Attention, CDR-SB, the TUG test, and International Shopping List Test-Recognition (a measure of working memory); in contrast, the 46% of participants with elevated plasma ptau181 showed minimal improvement over placebo.

While the clinical results will need to be confirmed in a hypothesis-testing clinical trial, we believe that, together, the two publications demonstrate translation of the preclinical findings to the clinic. The results also provide validation of our pathogenic model of BFC degeneration and of the Ts2 DS mouse as a translational platform for drug discovery and development, including target validation. This murine model can, therefore, provide preclinical proof-of-concept for novel therapeutic approaches to treating BFC dysfunction and degeneration [1].

Beyond the immediate conclusions, when the two reports are evaluated in the context of the broader scientific literature, their findings either confirm or are consistent with recent translational observations regarding BFC degeneration. *First*, as the magnitude of the treatment effect seen in the clinical study is greater than those reported with AChEI therapy and, notably, was seen on top of AChEI therapy, the study results strongly suggest that correcting the underlying physiologic defect yields greater treatment effects than does AChEI therapy. This result is consistent with recent evidence that optogenetic enhancement of BFCN function leads to physiologic release patterns that are expected to compensate more effectively for BFC dysfunction than the continuous elevations induced by cholinesterase inhibitors [4]. *Second*, neflamapimod was beneficial in both a preclinical DS model (Ts2 mice) phenocopying endosomal and cholinergic abnormalities of Early Onset AD [3] and in a clinical study in patients with DLB. These findings support the proposal that DLB and AD share a common Rab5-dependent pathogenic mechanism with respect to BFC degeneration, an idea consistent with emerging evidence that APP and alpha-synuclein have additive effects in driving degeneration in the basal forebrain [5–7]. In addition, in a translational clinical study examining associations between antemortem MRI brain scans and postmortem neuropathology in AD, basal forebrain atrophy was associated with both amyloid and Lewy body pathology, more strongly with the latter [8]. *Third*, the clinical results, particularly as reported in the second publication, are consistent with MRI studies that basal forebrain atrophy precedes and may be a driver of medial temporal lobe (hippocampal) atrophy in both AD [9] and DLB [2, 10]. From a therapeutics development standpoint these findings suggest that approaches targeting BFC degeneration are best applied in patients before they develop extensive hippocampal degeneration which, otherwise, would limit the therapeutic response (though such treatment may slow further disease progression in those with established hippocampal atrophy). Conversely, there is a strong rationale for combining approaches that exclusively target neurodegeneration in the hippocampus with approaches that target cholinergic degeneration in the basal forebrain.

## Data Availability

Not applicable

## Abbreviations

AD: Alzheimer’s disease
AChEI: acetylcholinesterase inhibitor
β-CTF: β-Secretase-cleaved carboxyl-terminal fragment of amyloid precursor protein
BFC: Basal Forebrain Cholinergic
CDR-SB: Clinical Dementia Rating Sum of Boxes
DLB: Dementia with Lewy bodies
TUG: Timed up and go
TID: Three-times-a-day
