# Correction: Drug development targeting degeneration of the basal forebrain cholinergic system: its time has come

**DOI:** 10.1186/s13024-023-00678-5

**Published:** 2023-11-09

**Authors:** John J. Alam, Ralph A. Nixon

**Affiliations:** 1 CervoMed Inc, 20 Park Plaza, Suite 424, Boston, MA 02116 USA; 2 Center for Dementia Research, Nathan S. Kline Institute for Psychiatric Research, Orangeburg, NY USA; 3 Departments of Cell Biology and Psychiatry, NYU Langone Medical Center, NYU Neuroscience Institute, New York, NY USA

**Molecular Neurodegeneration (2023) 18:74**

10.1186/s13024-023-00663-y

In the original article [1] the following statement of funding acknowledgement was mistakenly omitted:

“Preclinical studies have been supported by NIH grants P01AG017617 and R01AG062376 grants to RAN.”

